# Lower Third Molar Germectomy: Timings, Indications, and Clinical and Patient-Reported Outcomes—A Systematic Review and Meta-Analysis

**DOI:** 10.3390/dj14040215

**Published:** 2026-04-07

**Authors:** Francesco Scilla, Giulia Malvicini, Stefano Parrini, Nicola Baldini, Gioele Gioco, Sergio Mazzoleni, Stefano Sivolella, Simone Grandini

**Affiliations:** 1 Department of Head, Neck and Sense Organs, School of Dentistry, Catholic University of Sacred Heart, Fondazione Policlinico Universitario “A. Gemelli”-IRCCS Rome, 00135 Rome, Italy; francesco.scilla@unicatt.it (F.S.); gioele.gioco@unicatt.it (G.G.); 2 Oral Surgery Postgraduate School, Department of Medical Biotechnologies, University of Siena, 53100 Siena, Italy; stefano.parrini@unisi.it (S.P.); baldini.nicola@gmail.com (N.B.); 3 Unit of Endodontics and Restorative Dentistry, Department of Medical Biotechnologies, University of Siena, Viale Mario Bracci 11, 53100 Siena, Italy; simone.grandini@unisi.it; 4 Department of Endodontics, Rey Juan Carlos University, 28032 Madrid, Spain; 5 Department of Neurosciences, School of Dentistry, University of Padua, 35128 Padua, Italy; sergio.mazzoleni@unipd.it (S.M.); stefano.sivolella@unipd.it (S.S.)

**Keywords:** clinical indications, complications, germectomy, meta-analysis, oral surgery, outcomes, systematic review

## Abstract

**Background/Objectives**: To evaluate whether mandibular third molar germectomy is associated with differences in neurosensory injury, postoperative complications, and patient-centred outcomes compared with delayed extraction. **Methods**: A systematic review and meta-analysis were conducted according to PRISMA guidelines. Electronic searches were performed in PubMed, Embase, Web of Science, Scopus, Cochrane Library, LILACS, and Google Scholar. Comparative studies including pediatric or adolescent patients undergoing mandibular third molar germectomy were included. Primary outcomes were inferior alveolar nerve (IAN) injury and overall postoperative complications; secondary outcomes included postoperative morbidity and patient-reported outcome measures (PROMs). Random-effects models were used for quantitative synthesis. **Results**: Ten studies met the inclusion criteria, including two randomized controlled trials and eight observational studies. Comparative evidence suggested that early removal at an immature developmental stage was associated with a lower risk of IAN injury (pooled RR = 0.05, 95% CI 0.01–0.37) and fewer overall postoperative complications (pooled RR = 0.29, 95% CI 0.20–0.42) compared with delayed extraction. However, certainty of evidence was limited due to heterogeneity, risk of bias, and potential confounding. PROMs consistently showed a short-term increase in pain and temporary quality-of-life impairment after germectomy, while delayed-onset infections were reported several weeks after surgery. **Conclusions**: Germectomy may reduce neurosensory risk and overall postoperative morbidity in selected developmental-age patients but entails a measurable short-term symptom burden. Decisions should therefore rely on individualized risk assessment and shared decision-making rather than routine preventive removal. Further prospective comparative studies with standardized outcome reporting are required to support definitive clinical recommendations.

## 1. Introduction

Mandibular third molar management remains one of the most debated topics in oral surgery, particularly in adolescents and young adults in whom third molars are still developing and clinical decisions are often guided by preventive considerations rather than established disease [1,2]. Within this context, germectomy refers to the removal of a developing third molar at the rudimentary or early calcification stage, when it is already possible to anticipate that the tooth will not have sufficient space to erupt normally [3,4]. The procedure is commonly discussed in the context of children and adolescents, and some authors have suggested that earlier developmental stages may offer technical advantages because the third molar germ is typically located closer to the bone surface [3]. However, in both clinical practice and research, the timing of germectomy is more appropriately defined by radiographic developmental stage than by chronological age alone. This anatomical condition may make surgery technically simpler and less traumatic than extraction of fully formed, impacted mandibular third molars in adults [5].

The indications for germectomy are heterogeneous and often reflect a combination of orthodontic, infectious/cariogenic, and prophylactic rationales [3]. From an orthodontic perspective, removal of the developing third molar may be performed to facilitate molar distalization, reduce crowding-related concerns, or prevent future impaction patterns that could complicate orthodontic mechanics [6]. Clinically, germectomy is also considered in scenarios where the third molar germ is suspected to contribute to recurrent pericoronitis, distal caries or periodontal compromise of the second molar, or local inflammatory changes [3]. Finally, prophylactic germectomy has been advocated to prevent later pathology such as cystic change, tumor development, or progressive impaction [7,8]; however, the evidence base supporting routine prophylactic removal in developmental age remains limited, and the appropriateness of this practice is frequently dependent on patient-specific factors, local anatomy, and clinician experience.

A key clinical driver behind early removal is the goal of limiting neurological complications, particularly inferior alveolar nerve (IAN) and lingual nerve injury, which are among the most feared adverse events of mandibular third molar surgery [9,10,11]. The rationale is that earlier developmental stages may entail a different anatomic relationship between the developing third molar and surrounding neurovascular structures, potentially reducing the risk of permanent sensory disturbances [10]. Nevertheless, neurological risk is only one component of the overall harm–benefit profile: even if major nerve injury is rare, germectomy may still be associated with clinically relevant postoperative sequelae [11].

Indeed, germectomy can lead to short-term morbidity including pain, swelling, trismus, wound dehiscence, infection, and alveolar osteitis, with most events being transient but still impactful for adolescents and their caregivers [12].

Evidence on the optimal timing of mandibular third molar removal remains limited by heterogeneity in study populations, anatomical focus, and outcome definitions. As a result, the effect of early removal on neurosensory safety, postoperative complications, and recovery has not been clearly established.

Previous systematic reviews have addressed the management of mandibular third molars in developmental-age patients, focusing primarily on clinical indications and qualitative comparisons of outcomes. Staderini et al. [13] included only four studies and explicitly acknowledged that a quantitative analysis was not possible due to heterogeneity and limited data, while Mazur et al. [14] similarly restricted their conclusions to a qualitative synthesis of indications without providing pooled effect estimates for clinically relevant outcomes. Notably, neither review performed a meta-analysis nor provided quantitative data on inferior alveolar nerve injury or overall complication rates. The present systematic review and meta-analysis was therefore designed to address this gap, offering for the first time pooled effect estimates for these outcomes and enabling a more objective, evidence-based comparison between germectomy and delayed third molar removal.

Consequently, key clinical questions that directly influence surgical decisions, such as whether mandibular third molar germectomy is associated with a different risk of inferior alveolar (IAN) or lingual nerve injury compared to delayed extraction and whether it impacts postoperative complication rates relative to delayed intervention or observation, remain insufficiently clarified.

The present systematic review and meta-analysis, therefore, aims to provide a focused synthesis of the available evidence on mandibular third molar germectomy, with particular emphasis on outcomes most relevant to both patients and surgeons. Specifically, neurosensory injury, postoperative complications including delayed-onset infection, patient-centered recovery outcomes, surgical techniques, and follow-up strategies.

## 2. Materials and Methods

This systematic review was performed following the Preferred Reporting Items for Systematic Reviews and Meta-Analyses (PRISMA) statement (File S1) and the guidelines from the Cochrane Handbook for Systematic Reviews of Interventions [15]. The protocol was registered a priori in PROSPERO (1295710).

### 2.1. Eligibility Criteria

Eligibility criteria were defined a priori according to a PICO framework. We included studies enrolling patients with mandibular third molars in development (tooth germ/early root formation) undergoing one or two mandibular third molar germectomy, and reporting outcomes compared with either delayed mandibular third molar extraction performed at a more advanced stage of root development and/or older age, or watchful waiting/retention with extraction only if indications arose.

Eligible study designs were randomized controlled trials and comparative observational studies (prospective or retrospective cohort studies, and case–control studies) with no restrictions on publication language or date. We excluded case reports and non-comparative case series, narrative reviews/editorials, studies not providing a clear definition or timing for germectomy, and studies in which mandibular-specific data could not be separated from other sites or interventions.

In the present review, germectomy was operationally defined as the surgical removal of third molars at an early stage of development, typically corresponding to incomplete root formation (e.g., root formation ≤ 1/3; Nolla stage ≤ 7).

The term “early extraction” was considered in a broader sense in the included studies. When necessary, studies using different terminology were carefully evaluated to ensure conceptual consistency with the defined intervention.

Early removal was defined based on developmental stage, including incomplete root formation and early Nolla stages. In this review, germectomy refers to the removal of a developing third molar at an early stage, whereas early extraction with incomplete root formation may include a broader range of developmental stages.

Due to the limited availability of comparative studies addressing the timing of third molar removal, observational single-arm studies were also considered eligible for inclusion in the qualitative synthesis when they provided relevant data on postoperative complications following germectomy.

However, only studies including a comparator group (early vs. delayed extraction) were considered eligible for quantitative synthesis (meta-analysis).

### 2.2. Search Strategy and Study Selection

Literature searches were conducted using a combination of MeSH terms and free-text keywords in PubMed (MEDLINE), Google Scholar, Cochrane Library and Scopus, with no time limit. The detailed search strategy reported below was developed for PubMed, while corresponding strategies were adapted for the other databases in accordance with their specific syntax and indexing systems. All searches combined subject headings with free-text terms. The final search strategy was refined after multiple pre-searches. The search string was as follows: ((“Molar, Third”[Mesh] OR “third molar*”[tiab] OR “wisdom tooth*”[tiab] OR “wisdom teeth”[tiab] OR “3rd molar*”[tiab] OR “third-molar*”[tiab]) AND (“Tooth, Impacted”[Mesh] OR “Tooth, Unerupted”[Mesh] OR impacted[tiab] OR impaction[tiab] OR unerupted[tiab] OR ((germ[tiab] OR “tooth germ*”[tiab] OR “tooth bud*”[tiab] OR “dental germ*”[tiab]) AND (“third molar*”[tiab] OR “wisdom tooth*”[tiab] OR “wisdom teeth”[tiab] OR “3rd molar*”[tiab] OR “third-molar*”[tiab]))) AND (“Tooth Extraction”[Mesh] OR extraction[tiab] OR extract*[tiab] OR remov*[tiab] OR odontectomy[tiab]) AND (indication*[tiab] OR prophylactic[tiab] OR early[tiab] OR timing[tiab] OR delayed[tiab] OR “watchful waiting”[tiab] OR retention[tiab] OR monitoring[tiab] OR “conservative management”[tiab])) OR (germectomy[tiab]).

Cross-checking the reference lists of primary research reports was done to find additional studies. In accordance with the inclusion criteria, two authors (F.S. and G.M.) independently selected articles based on titles and abstracts using Raayaan (Rayyan Systems, Inc., Cambridge, MA, USA). The full texts of the selected articles were then read to assess their suitability for inclusion. Disagreements were resolved through consensus or by consulting a third author (N.B.).

The last literature search was conducted on 31 January 2026, ensuring that the most recent available evidence was included in the review.

### 2.3. Data Collection

The data were first extracted independently and in duplicate by the same reviewers (FS and GM) using specially designed data extraction forms. For studies matching with the inclusion criteria, or for which information in the title and abstract was insufficient to make a clear decision, the reviewers obtained and screened the full report.

### 2.4. Data Items

The following data were extracted from each included study: study design and setting, sample size, patient characteristics (including age and sex), number of patients and number of mandibular third molars treated, developmental stage at the time of intervention, type and timing of surgical intervention (germectomy or delayed extraction), comparator treatment when applicable, reported clinical indications, postoperative complications (including neurosensory injury and infectious events), PROMs when available, and duration and completeness of follow-up.

### 2.5. Outcomes Definition

Outcomes were defined a priori in accordance with the review objectives and PICO framework and were categorised as primary and secondary outcomes.

Primary outcomes were: (i) inferior alveolar nerve (IAN) injury, defined as any reported postoperative sensory disturbance within the distribution of the inferior alveolar nerve, including paresthesia, hypoesthesia, dysesthesia, anesthesia, or numbness, as assessed clinically or reported by the patient.

When available, IAN injury was further categorized according to duration as transient (resolution within the follow-up period) or persistent/permanent (lasting ≥6 months or reported as ongoing at the last follow-up); and (ii) overall postoperative complications, defined as a composite outcome including any reported adverse event following surgery (e.g., infection, alveolar osteitis, bleeding, swelling, trismus, wound dehiscence, or neurosensory disturbances), irrespective of whether individual studies formally defined these as complications.

Secondary outcomes included: (i) postoperative morbidity parameters, such as pain (e.g., visual analogue scale [VAS]), swelling, trismus (mouth opening limitation), and functional impairment; and (ii) patient-reported outcome measures (PROMs), including quality-of-life instruments (e.g., OHIP-14, SF-12) and subjective recovery trajectories.

### 2.6. Quality Assessment

Two reviewers independently extracted and appraised outcomes using a standardized data-collection form. Methodological quality was rated using predefined, review-specific criteria, and the certainty of the evidence for each study was classified as high, moderate, or low. A supplemental analysis was performed independently by the two examiners regarding the overall quality of the evidence for any performed meta-analysis using the Grading of Recommendations, Assessment, Development, and Evaluations (GRADE) system.

### 2.7. Risk of Bias in Individual Studies

Risk of bias was assessed at the study level using design-specific tools. Non-randomized studies were evaluated with ROBINS-I, whereas randomized controlled trials were assessed using the Cochrane Risk of Bias 2 (RoB 2) tool. Visual summaries were generated using the robvis tool [16].

### 2.8. Meta-Analysis

Quantitative synthesis was performed when at least two studies reported comparable outcomes.

Meta-analyses were conducted for inferior alveolar nerve injury and for overall postoperative complications. Effect sizes were expressed as risk ratios (RRs) and pooled on the logarithmic scale; results are presented as exponentiated estimates (RRs) for interpretability. Event counts and total sample sizes were extracted for each group and used to compute study-specific risk ratios. Given the expected clinical and methodological heterogeneity among studies, random-effects models were used as the primary analytical approach. The restricted maximum likelihood (REML) method was applied to estimate between-study variance. Random-effects models were used to address expected clinical and methodological heterogeneity, and fixed-effect models were also performed as sensitivity analyses to assess the robustness of the findings.

For outcomes with zero events in one or more study arms, a continuity correction of 0.5 was applied. Statistical heterogeneity was assessed using the I^2^ statistic and Cochran’s Q test.

Formal assessment of publication bias (e.g., funnel plots or regression-based tests) was planned only if a sufficient number of studies were available for a given meta-analysis, as these methods are not considered reliable when based on a small number of studies.

Meta-analyses were considered exploratory due to the limited number of comparative studies. When studies reported more than one early-treatment group, these were combined into a single early group for analysis. All analyses were conducted using Stata BE (version 18.05; StataCorp, College Station, TX, USA). A two-sided *p* value < 0.05 was considered statistically significant.

## 3. Results

### 3.1. Study Selection

The electronic search identified 1904 records. After removal of duplicates, 1056 records remained for title/abstract screening. A total of 44 full-text articles were selected. Following full-text evaluation, ten studies were included in the review.

**Figure 1:**
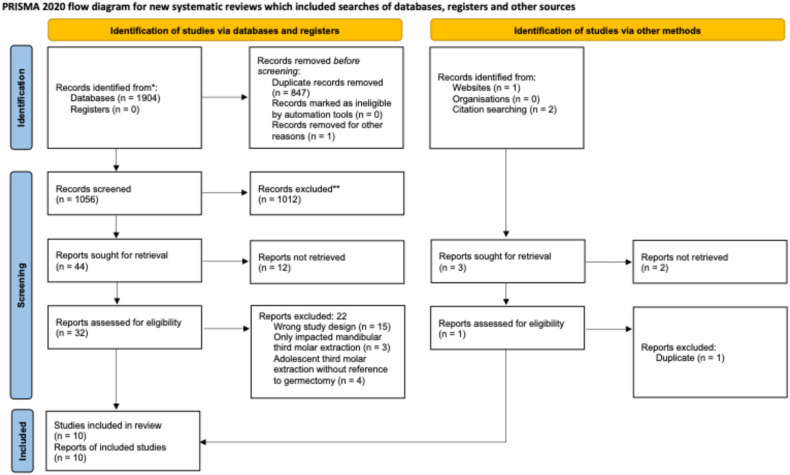
PRISMA flow diagram.

No additional publications were recovered after checking the bibliographies of all the selected articles. The study selection process is summarized in the PRISMA flow diagram (Figure 1).

### 3.2. Study Characteristics

#### 3.2.1. Excluded Studies

After full-text evaluation, 34 articles were excluded for the following reasons: 15 did not have a study design consistent with the predefined eligibility criteria; 8 had no retrievable full text, and the full texts could not be obtained even after contacting the authors; 4 studies were only narrative review of the topic; 3 investigated only extraction of impacted mandibular third molars; and 4 evaluated mandibular third molar extraction in adolescents without specific reference to germectomy.

#### 3.2.2. Included Studies

Among the included studies, several were observational single-arm studies without a comparator group. These studies were included to provide additional evidence on the safety profile and postoperative complications associated with germectomy.

However, they were not included in the quantitative synthesis, which was restricted to studies directly comparing early versus delayed third molar removal. Consequently, the meta-analysis was conducted on a subset of studies meeting the predefined PICO comparison, while the remaining studies contributed to the qualitative synthesis.

Most of the studies were carried out in Italy [12,14,16,17,18,19,20], one in France [11], one in Uzbekistan [21] and one in China [10].

All the studies included in the qualitative synthesis comprised pediatric and adolescent patients undergoing mandibular third molar germectomy and, in a subset of datasets, a comparator group treated with delayed extraction at a more advanced stage of root development. The design of the studies revealed three retrospective studies [10,12,16], five prospective observational studies [11,14,19,20,21], and two randomized controlled trials [17,18]. Characteristics of all the included studies are summarized in Table 1.

The most frequently reported complications of germectomies are displayed in Table 2.

Quantitative synthesis was feasible for inferior alveolar nerve injury based on two comparative studies.

### 3.3. Characteristics of Participants

The selected studies included children, adolescents, or young adults subjected to third molar germectomy according to a retrospective or prospective design.

In accordance with the aims of the present review, when studies reported mixed populations including adolescents and adults, we extracted and analyzed only the data pertaining to patients undergoing mandibular third molar germectomy during the developmental stage consistent with our definition of early removal and excluded data referring to fully developed third molars or standard third molar extraction in adults.

The timing of intervention was defined mainly by radiographic root development rather than chronological age. “Early” treatment generally corresponded to tooth buds/immature roots (e.g., incomplete root formation/apical closure), whereas “delayed” extraction typically reflected mature roots with closed apices or older age categories.

### 3.4. Characteristics of Interventions and Outcomes Measures

Across the included studies, the intervention was consistently defined as mandibular third molar germectomy/early removal performed at an immature developmental stage (before complete root formation), typically for orthodontic indications, while comparators, when present, were delayed extractions of fully developed third molars with closed apices/older age groups. Surgical protocols varied in technical details, but procedures were generally standardized within studies and often performed by a limited number of operators. Outcome assessment was heterogeneous but clustered around two domains: (i) safety/complications, including overall postoperative complications, inferior alveolar/lingual nerve injury, bleeding, and delayed-onset infection occurring weeks after surgery; and (ii) patient-centred morbidity, including pain trajectories (VAS), swelling, trismus/mouth opening, and quality-of-life measures at early follow-up (OHIP-14 or SF-12). Importantly, several studies reported complications in sufficient detail to support pooled analyses for IAN injury and overall postoperative complications, while other trials focused mainly on short-term morbidity metrics (pain/swelling/function) rather than discrete adverse-event reporting.

Across the 10 included studies, only some provided comparative data [10,16,21] between early removal and delayed extraction; therefore, quantitative synthesis was limited to a small number of outcomes. In the available comparative evidence, early removal was associated with a significantly lower risk of IAN injury (pooled RR = 0.05, 95% CI 0.01–0.37; 2 studies [10,16]) and with fewer overall postoperative complications (pooled RR = 0.29, 95% CI 0.20–0.42; 3 studies [10,16,21]), with no detectable heterogeneity in these pooled analyses. Notably, some non-comparative studies [12,17,19] highlighted that postoperative infection may present with delayed onset (up to 2–8 weeks), an aspect that may be underestimated when follow-up is restricted to the early postoperative period.

### 3.5. Quality Assessment (Risk of Bias)

**Figure 2:**
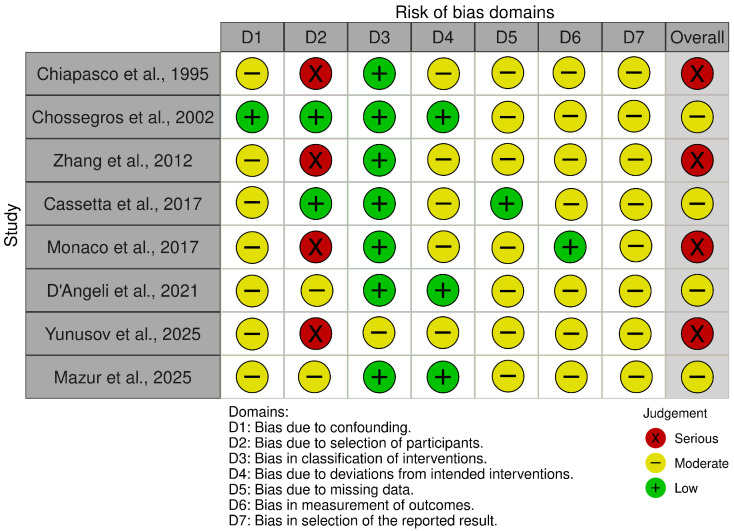
Risk of bias graph of non-randomized studies using the ROBINS-I tool [10,11,12,14,16,19,20,21].

**Figure 3:**
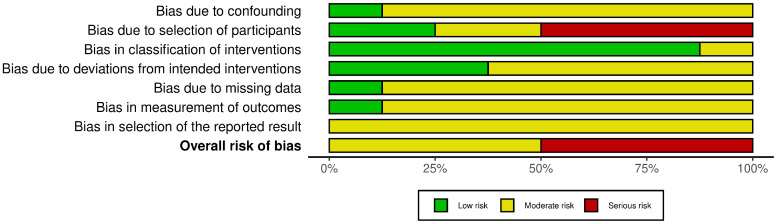
Risk of bias summary of non-randomized studies using the ROBINS-I tool.

**Figure 4:**
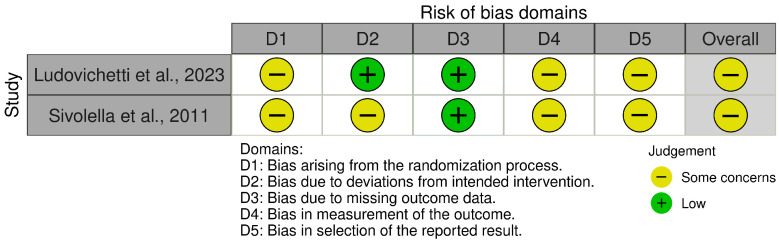
Risk of bias graph of randomized controlled trials using the Cochrane Risk of Bias 2 (RoB 2) tool [17,18].

**Figure 5:**
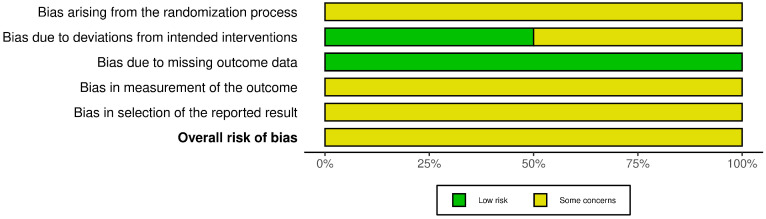
Risk of bias summary of randomized controlled trials using the Cochrane Risk of Bias 2 (RoB 2) tool.

The risk-of-bias assessments are summarized in Figure 2, Figure 3, Figure 4 and Figure 5. Because the review included both randomized and non-randomized studies, different tools were used according to study design. The eight non-randomized studies [10,11,12,14,16,19,20,21] were assessed with ROBINS-I, whereas the two randomized controlled trials [17,18] were evaluated separately using RoB 2.

Among the non-randomized studies, overall risk of bias was judged as moderate in five studies [11,14,19,20] and serious in three studies [10,16,21]; no study was judged at low overall risk. The most important source of bias concerned confounding/baseline differences, whereas classification of the intervention was generally judged at low risk, indicating that timing of removal was usually well defined. Concerns also frequently emerged in relation to missing data, outcome measurement, and selective reporting.

The two randomized studies were judged as presenting some concerns overall. In Ludovichetti et al. [18], concerns mainly related to insufficient reporting of allocation concealment and limited detail on pre-specified analysis, despite the absence of missing data and blinded outcome assessment. In Sivolella et al. [17], some concerns were mainly related to the lack of blinding and the inclusion of subjective postoperative outcomes, although random sequence generation was reported and completeness of outcome data appeared adequate.

Formal evaluation of small-study effects was not feasible because each meta-analysis included fewer than ten studies; therefore, publication bias could not be reliably assessed. The GRADE approach was used to rate the certainty of the evidence and the strength of the conclusions (Table 3). Although both meta-analyses were based on comparative cohort studies, the certainty of evidence was judged to be limited, mainly because of non-randomized study designs, concerns regarding confounding and risk of bias, and imprecision related to the small number of studies and, for rare outcomes, wide confidence intervals.

As a matter of fact, although two randomized controlled trials were included in the qualitative synthesis, they were not eligible for quantitative pooling and therefore did not contribute to the GRADE assessment.

### 3.6. Meta-Analysis

**Figure 6:**
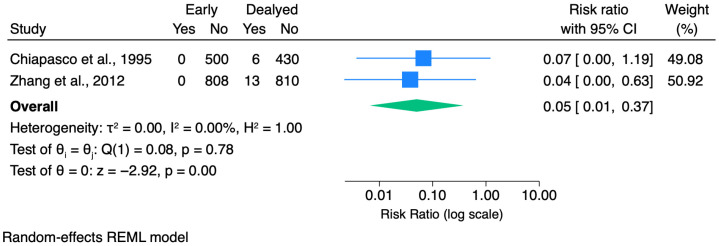
Forest plot of the risk of inferior alveolar nerve (IAN) injury comparing early mandibular third molar removal (germectomy) with delayed extraction [10,16]. Effect sizes are expressed as risk ratios (RRs) with 95% confidence intervals and are plotted on a logarithmic scale. Raw data (events and non-events) are displayed for each study. A random-effects REML model was used for pooling. Studies reporting zero events in one or more groups were handled using a continuity correction.

Pooled analysis using a random-effects model showed a significantly lower risk of IAN injury following early mandibular third molar removal compared with delayed extraction (pooled RR = 0.05, 95% CI 0.01–0.37, *p* = 0.003). Both studies demonstrated a consistent direction of effect favoring early removal, and no statistically detectable heterogeneity was observed (I^2^ = 0%) (Figure 6). Given the limited number of studies included, these results should be interpreted as exploratory. When multiple early-treatment groups were reported, these were combined into a single early group. In Yunusov et al., [21] Groups A and B were merged and compared with Group C (delayed extraction).

A random-effects model was used to account for expected between-study differences in both clinical and methodological features, including study design, how “early” versus “delayed” removal was defined, surgical protocols, and outcome assessment. In addition, because only two studies were available, heterogeneity statistics (e.g., I^2^) are unreliable and may fail to detect true variability. For these reasons, we consider random-effects pooling more appropriate than a fixed-effects model.

**Figure 7:**
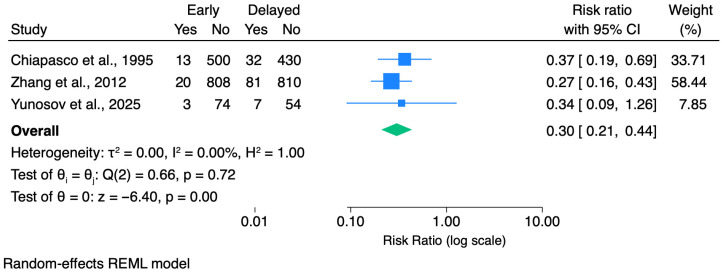
Forest plot of the risk of overall postoperative complications comparing early mandibular third molar removal (germectomy) with delayed extraction [10,16,21]. Effect sizes are expressed as risk ratios (RRs) with 95% confidence intervals and are plotted on a logarithmic scale. Raw data (events and non-events) are displayed for each study. A random-effects REML model was used for pooling. In Yunusov et al. [21], early developmental groups were combined and compared with the delayed-extraction group.

Three comparative studies were included in the meta-analysis of overall postoperative complications (Figure 7). Using a random-effects model, early mandibular third molar removal was associated with a significantly lower risk of overall postoperative complications compared with delayed extraction (pooled RR = 0.29, 95% CI 0.20–0.42, *p* < 0.001). All included studies showed a reduction in complication rates favoring early removal, although one study did not reach statistical significance individually. No statistically detectable heterogeneity was observed (I^2^ = 0%). Given the heterogeneous definitions of postoperative complications across studies and the limited number of available studies, findings should be considered exploratory. All results were consistent when a fixed-effect model was applied.

## 4. Discussion

This systematic review synthesized the evidence comparing germectomy/immature extraction with delayed extraction in pediatric and adolescent populations. Across the ten included studies, “early” intervention was primarily defined by radiographic developmental stage (tooth germ or immature roots), rather than chronological age alone, consistent with the biological premise that surgical difficulty, bone density and neurovascular proximity evolve with root maturation. The evidence base remains heterogeneous in design, reporting unit (patients vs. teeth/extractions), outcome definitions and follow-up schedules; nevertheless, selected endpoints were sufficiently comparable to support exploratory quantitative synthesis for IAN injury and overall postoperative complications.

### 4.1. Early Versus Delayed Extraction: Neurosensory Safety

Given the heterogeneity in definitions and reporting across studies, all reported IAN sensory alterations were aggregated into a single binary outcome (presence/absence of IAN injury) for quantitative synthesis. When multiple timepoints were reported, the longest available follow-up was considered to capture persistent events.

The most clinically consequential comparative endpoint was IAN injury, meta-analyzed using two comparative cohorts [10,16]. The definition of IAN injury varied across studies, including terms such as paresthesia, dysesthesia, and numbness. Most reported cases were transient, with only a few persistent sensory disturbances described, such as one case lasting 25 months in Chiapasco et al. [16] and two cases exceeding 6 months in Zhang et al. [10].

The pooled estimate strongly favored early removal, and both studies were directionally consistent with a maturity-dependent risk gradient: Chiapasco et al. [16] observed that complication rates increased in progressively more advanced groups, including a prolonged paresthesia in the most advanced group; similarly, Zhang et al. [10] reported no nerve injury in the immature group and higher complication rates, including nerve lesions, in the mature/delayed group. This finding is consistent with, and further supports, conclusions previously reported in the literature [9,22,23].

Importantly, this finding should not be interpreted as evidence of a novel or unexpected protective effect specific to germectomy, but rather as a quantitative confirmation, within a germectomy-focused evidence base, of a maturity-dependent neurovascular risk gradient that is already well established in the broader third molar literature, rather than as evidence of a novel protective effect specific to germectomy.

The anatomical rationale is straightforward: as root formation progresses, the apex migrates closer to the inferior alveolar canal, the periodontal ligament narrows, bone density increases, and surgical complexity rises, all factors independently associated with higher rates of nerve injury and less complete recovery [5,24].

In the comparative studies included in the present meta-analysis, “early” removal consistently corresponded to incomplete root formation with open apices and greater tooth-to-canal distance, whereas “delayed” extraction involved fully formed roots, closed apices, and older age groups, precisely the conditions under which neurosensory risk is known to be higher. The lower risk of IAN injury observed with germectomy is therefore biologically coherent and directionally expected. It should nonetheless be interpreted with caution, given the small number of comparative studies, the non-randomised designs, the risk of confounding by indication, and the absence of standardised neurosensory testing across included studies.

With respect to the lingual nerve, morbidity appears uncommon in true germectomy cohorts. A large prospective randomized study assessing lingual nerve protection during germectomy reported no subjective or objective evidence of transient or permanent lingual nerve injury, suggesting that routine protection may not be necessary under standardized conditions [11]. However, neurosensory outcomes were not consistently assessed using standardized testing or long follow-up across studies, so transient disturbances may be under-detected when evaluation relies on routine clinical reporting.

### 4.2. Overall Postoperative Complications

Meta-analysis of overall postoperative complications (Chiapasco et al. [16], Zhang et al. [10], and Yunusov et al. [21]) suggested fewer complications with early removal. While clinically encouraging, this result should be interpreted cautiously because “overall complications” were typically reported as a composite endpoint with heterogeneous event definitions (e.g., infection, alveolar osteitis, bleeding, swelling, trismus and neurosensory symptoms) and variable ascertainment windows. In Yunusov et al. [21], complications were lower in developmental-age groups than in an adult comparator group; however, limited reporting of specific complication types constrained clinical interpretation. These limitations highlight the need for future comparative studies to report predefined complication categories separately and adopt harmonized follow-up schedules to improve interpretability and enable robust pooling.

### 4.3. Infection and the Late Window

A clinically relevant nuance is the potential for delayed-onset infection beyond conventional early reviews. Monaco et al. [12] documented delayed infections in 20 of 218 germectomies (9.2%), with onset between 2 and 8 weeks after surgery, and found that reduced distal space behind the second molar was significantly associated with this complication. In 16 of the 20 infected cases, the Ganss ratio was <0.5, supporting an anatomical mechanism related to food stagnation and impaired distal cleansing rather than to immediate postoperative inflammation alone.

D’Angeli et al. [19] also reported a delayed-onset infection at 4 weeks in a patient treated at Nolla stage 7, further supporting the existence of a late infectious window after germectomy. From a clinical perspective, these findings suggest that follow-up protocols restricted to the first postoperative week are likely to underestimate this outcome, and that patients and caregivers should be specifically counselled regarding the possibility of late swelling, purulent discharge, or discomfort after apparently uneventful early healing.

### 4.4. Surgical Invasiveness and Healing-Related Sequelae

Ludovichetti et al. [18] addressed flap design specifically in the germectomy context. In a double blind randomised clinical trial (n = 40 patients aged 11–16 years; 40 germectomies; marginal vs. para marginal flap), the para-marginal approach produced significantly better outcomes across all measured parameters: plaque index (mean 3.85% vs. 13.3%, *p* = 0.001), bleeding on probing (30% vs. 95% post-surgical positive, reduction of ~65%), distal probing depth increase (0.4 ± 0.69 vs. 1.3 ± 0.47 mm, *p* = 0.00003), postoperative pain (VAS 2.95 ± 1.93 vs. 5.75 ± 1.37, *p* < 0.00001), and swelling (55% absence vs. 0% absence in marginal group). The para-marginal incision preserves the supracrestal tissue attachment and keratinised gingival collar around the second molar, prevents papillary disruption, and reduces plaque-retentive anatomy distal to the second molar.

Notably, Chiapasco et al. [16] also used a triangular para-marginal flap in their germectomy group (Group A), and recorded no nerve damage or alveolar osteitis, lending further support to this approach. Sivolella et al. [17] noted that wound dehiscence occurred in approximately 27–31% of cases at 7 days regardless of osteotomy technique, but resolved completely by 30 days with formation of a fibromucosal pad. The authors linked the pattern of dehiscence to flap design rather than to the osteotomy instrument used, consistent with the findings of Ludovichetti et al. [18].

The contextual evidence from Pogrel et al. [5] documents that periodontal healing distal to the second molar is strongly age-dependent. About this topic, Kugelberg et al. [25] showed that none of the patients under 25 years developed worsening intrabony defects, while 29.6% of older patients did.

### 4.5. Patient-Centred Outcomes and Recovery

Mazur et al. [3] provides the most granular patient-reported outcome data currently available for germectomy in the paediatric population. Mean VAS pain scores followed a monotonic decline from approximately 5.9 at T1 to 0.54 at T7. Longer operative time was the only factor significantly associated with delayed pain recovery at postoperative day 3 (OTD coefficient 0.00128 per second, *p* = 0.069), confirming that surgical efficiency directly influences postoperative morbidity. The subjective pain threshold did not increase significantly between a first and second germectomy (mean SPT 4.3 ± 2.0 vs. 4.6 ± 2.4, *p* = 0.560), indicating that adolescents do not undergo systematic sensitisation between staged bilateral procedures. Patients with higher self-rated health showed significantly lower pain scores at T1 (Pearson r = −0.32, *p* < 0.05), underscoring the role of psychosocial factors in recovery, a finding with practical implications for preoperative counselling.

D’Angeli et al. [19], in a pilot clinical trial with 2-year follow-up (n = 25 patients, mean age 15.44 ± 2.06 years; 46 germectomies, Nolla stages 5–8), reported a complication prevalence of 4.3% (2/46 procedures, 8% of patients): one delayed-onset infection at 4 weeks (Nolla stage 7) and one immediate postoperative haemorrhage (Nolla stage 6). No cases of swelling, trismus, alveolar osteitis, or neural paresthesia were recorded at any follow-up visit (1 week, 2 weeks, 1 month, 1 year, 2 years).

The authors attributed the very low complication rate to the age-related advantage (adolescents recover faster), standardised surgical technique, and the combined suture protocol tested in this study (oblique interrupted plus Donati mattress suture), which provided stable flap closure distal to the second molar and prevented gingival hypertrophy.

Sivolella et al. [17], in a randomised prospective crossover trial (n = 26 patients, mean age 15.4 ± 1.29 years; 52 germectomies comparing piezoelectric vs. rotatory osteotomy), found no statistically significant between-group differences in pain (VAS), mouth opening, soft tissue appearance, lymphadenopathy, wound dehiscence, or persistent oedema at 7 and 30 days (all *p* > 0.05). The piezoelectric technique took significantly longer (15.77 vs. 11.77 min total procedure time; *p* = 0.028), which likely neutralised its theoretically lower tissue trauma. All variables were comparable between groups at 30 days, consistent with the rapid functional recovery typical of adolescent patients. Wound dehiscence was observed in 26.9–30.8% of cases at 7 days but resolved by 30 days, with formation of a fibromucosal pad distal to the second molar, a finding with direct relevance to periodontal outcomes.

Yunusov and Sultanov [21], comparing germectomy in two paediatric age groups (Group A: 9–12 years, n = 16, 28 teeth; Group B: 13–17 years, n = 20, 46 teeth) vs. fully formed impacted molar extraction in adults (Group C: ≥18 years, n = 32, 54 teeth), reported complication rates of 3.6%, 4.3%, and 13.0% respectively (between-group difference *p* > 0.05, though the trend towards higher rates in Group C is clinically meaningful given the sample size). The authors recommend germectomy between 11 and 15 years, noting that patients ≤11 years often lack sufficient cooperation for local anaesthesia procedures, while patients >17 years have near-complete root calcification that raises surgical difficulty.

Beyond nerve-related complications, germectomy is associated with postoperative morbidity, including pain, swelling, and trismus, which represent clinically relevant outcomes from a patient-centred perspective. Available evidence suggests that these symptoms are generally transient but may significantly affect short-term quality of life, particularly in younger patients.

PROMs consistently indicate a temporary impairment in daily activities and oral function during the early postoperative period. Although these effects tend to resolve within a few days, their clinical relevance should not be underestimated when considering the timing of third molar removal.

### 4.6. Effect Modifiers, Indications and Confounding

Several studies suggest that postoperative recovery is influenced not only by timing but also by technique, protocols, and operator factors (e.g., piezoelectric vs. rotatory osteotomy), which plausibly contribute to between-study variability [17,26]. A persistent methodological limitation is confounding by indication: early removal is often performed for orthodontic planning or prophylaxis, whereas delayed extraction is frequently symptom-driven and undertaken in the presence of mature impaction and higher baseline complexity [13]. Consistent with this, the risk-of-bias assessment identified concerns regarding baseline differences/confounding, and incomplete capture of late events, in several studies. Therefore, while the direction of findings is clinically coherent, certainty remains limited and pooled estimates should be considered exploratory.

### 4.7. Clinical Implications

Overall, the available evidence supports considering early mandibular third-molar removal in selected developmental-age patients when the primary objective is to minimize neurosensory risk and potentially reduce postoperative morbidity, while explicitly counseling about short-term symptoms, QoL impact and the possibility of late infection. In practical terms, germectomy may be most justifiable when an immature radiographic stage coexists with a credible trajectory toward impaction or second-molar interference, and when the anticipated benefit outweighs the short-term morbidity and follow-up requirements. From a surgical perspective, early removal does not necessarily imply a minimally invasive procedure. In early developmental stages, the tooth germ may still be partially or completely embedded within bone, often requiring flap elevation, osteotomy, and surgical exposure. These factors may influence postoperative discomfort and healing dynamics, potentially contributing to local bone remodeling and transient morbidity. Therefore, the surgical complexity of germectomy should not be underestimated and may vary depending on anatomical conditions and operator experience.

Periodontal outcomes distal to the second molar represent an additional clinically relevant aspect of third molar surgery. Previous studies [25,27] have shown that third molar removal may be associated with periodontal defects, particularly in adult patients. In this context, earlier intervention may be associated with more favorable periodontal healing due to reduced root development and improved regenerative potential.

Another important consideration is the occurrence of delayed-onset infections, which may not be captured in short-term follow-up. These complications have been associated with factors such as reduced distal space, food impaction, and impaired plaque control. Their reported incidence varies across studies, but they may have implications for postoperative monitoring and follow-up strategies.

Overall, different postoperative complications, including infection, swelling, and trismus vary in timing, severity, and clinical relevance, and should be interpreted within their specific clinical context. These findings are consistent with previous literature on complications of third molar surgery [24,28].

### 4.8. Key Findings

In limited comparative evidence, early removal favors reduced IAN injury and fewer overall postoperative complications compared with delayed extraction (RR 0.05 and RR 0.29, respectively) [10,16,21].

Evidence certainty is limited due to non-randomized designs, few comparative studies and heterogeneity in definitions/ascertainment; pooled estimates indicate direction rather than definitive effect size.

Delayed-onset infection weeks after surgery is clinically relevant and should be incorporated into counseling and follow-up planning [12].

### 4.9. Strengths and Limitations

A key strength of this review is the protocol-driven focus on mandibular germectomy/immature extraction and clinically relevant patient-facing outcomes, including late infectious events [12].

Limitations include a small comparative evidence base, heterogeneity in staging and outcome definitions, and susceptibility to confounding by indication [13]; PROMs and standardized neurosensory testing were applied inconsistently [11,14]. Higher-quality prospective comparative studies with harmonized core outcome sets are needed to support definitive recommendations.

A limitation of this review is the use of a composite outcome defined as overall postoperative complications. Early and delayed complications may differ substantially in pathogenesis and clinical relevance, as illustrated by the delayed-onset infection pattern documented by Monaco et al. (2017) [12], characterized by a distinct anatomical predictor (Ganss ratio <0.5) and onset 2–8 weeks after surgery, clearly distinct from immediate complications. This composite approach was adopted solely because none of the comparative studies stratified complications by temporal onset, precluding separate pooling. Individual complication types were extracted and reported separately in the qualitative synthesis whenever data permitted. Future comparative studies should pre-specify complication categories (e.g., immediate: haemorrhage, alveolar osteitis; delayed: infection, wound breakdown) with defined ascertainment windows of at least 8 weeks, to enable robust and clinically meaningful pooling.

Another limitation is the inclusion of observational single-arm studies that do not fully adhere to the predefined PICO framework. However, these studies were retained in the qualitative synthesis as they provide valuable information on complication rates and risk factors associated with germectomy, which remain poorly investigated in the current literature. Their exclusion would have resulted in a substantial loss of clinically relevant information.

Additionally, the study by Sivolella et al. [17] primarily evaluated surgical technique rather than the timing of extraction. Although not strictly aligned with the intervention-comparison structure, it was included as it provides relevant data on postoperative morbidity following germectomy and contributes to the overall understanding of the procedure.

Considering the limited number of comparative studies and the overall methodological limitations of the available literature, the present systematic review does not allow definitive, absolute indications or contraindications for routine preventive mandibular third-molar germectomy in pediatric and adolescent patients. The certainty of evidence remains limited, primarily due to non-randomized designs, the risk of confounding, and imprecision arising from a few studies and rare events.

Despite these limitations, the comparative evidence suggests that early removal at an immature developmental stage may be associated with clinically relevant benefits in selected patients, particularly in terms of neurosensory safety and, more broadly, the burden of postoperative morbidity. In exploratory meta-analyses, early removal was associated with a markedly lower risk of IAN injury compared with delayed extraction, with observational comparative cohorts consistently reporting higher nerve injury rates in older/more mature extraction groups [10,16].

Likewise, the overall postoperative complication rate tended to be lower after early removal in comparative cohorts, although this endpoint represents a composite outcome with heterogeneous definitions and inconsistent follow-up windows across studies; therefore, pooled estimates should be interpreted as indicative of direction rather than definitive effect size [10,16,19].

Furthermore, the observed reduction in nerve injury should be interpreted as a confirmation of known anatomical and developmental factors rather than as evidence of a novel protective effect. Finally, several clinically relevant aspects of germectomy, including postoperative morbidity, surgical invasiveness, periodontal outcomes, and long-term benefits, remain incompletely explored and warrant further investigation.

Clinical decision-making should follow a problem-oriented, patient-centered approach, integrating clinical and radiographic assessment with the patient’s orthodontic trajectory, preferences, and expected compliance. Importantly, clinicians should counsel adolescents and caregivers that germectomy is not a “zero-morbidity” procedure and may be associated with short-term symptoms and quality-of-life impairment, while a clinically relevant proportion of infections may present with delayed onset weeks after surgery, an event that can be missed if follow-up is restricted to the first postoperative week [12,19].

Future research should prioritize prospective comparative cohorts or pragmatic trials using harmonized definitions of early versus delayed removal based on developmental staging, standardized neurosensory assessment with time-to-recovery reporting, and pre-specified complication categories (including delayed-onset infection), alongside a core set of patient-reported outcomes measured at agreed timepoints [12,20].

### 4.10. Novelty

The present study advances the existing literature by moving beyond the qualitative syntheses that have characterised previous systematic reviews on this topic. Both Staderini et al. [13] and Mazur et al. [14] provided valuable descriptive overviews of clinical indications for germectomy, but neither was able to perform a quantitative synthesis: Staderini et al. explicitly stated that a meta-analysis was precluded by the heterogeneity and limited number of included studies, while Mazur et al. confined their conclusions to a narrative assessment of indications. In contrast, the present review provides pooled effect estimates for two clinically relevant primary outcomes, inferior alveolar nerve injury and overall postoperative complications, thereby offering a more objective and clinically actionable assessment of the risk–benefit profile of early versus delayed third molar removal. Importantly, the quantitative approach also exposes the gap between widely accepted clinical assumptions and the actual strength of the available evidence, underscoring the need for well-designed prospective trials in this field.

## 5. Conclusions

Mandibular third molar germectomy may offer advantages in selected developmental-age patients, particularly in reducing neurosensory risk and overall postoperative complications compared with delayed extraction. However, the available evidence remains limited by methodological heterogeneity and potential confounding. Germectomy should therefore not be considered a routine preventive procedure, as it is associated with a measurable short-term postoperative burden. Clinical decisions should rely on individualized risk assessment and shared decision-making until higher-quality comparative evidence becomes available.

## Figures and Tables

**Table 1:** Study characteristics of the included studies.

Author (Year)	Study Design	Country/Setting	Population (n of Patients; n. of Teeth; Age)	Tooth Development Stage/Timing	Intervention	Comparator	Follow-Up	Main Clinical Outcomes
Chiapasco et al., 1995 [16]	Retrospective cohort	Italy/University clinic	254 pts; 1500 teeth; 9–16 y	Immature mandibular third molars	Mandibular third molar germectomy	Delayed extraction (≥24 y)	Not specified	Overall complications; nerve injury
Monaco et al., 2017 [12]	Prospective cohort	Italy/University clinic	134 pts; 218 teeth; 12–20 y	Germ stage	Mandibular germectomy	None	≥12 months	Delayed-onset infection
D’Angeli et al., 2021 [19]	Prospective cohort	Italy/University clinic	25 pts; 46 teeth; mean 15.4 y	Nolla stage 6–7	Mandibular germectomy	None	Up to 24 months	Postoperative complications
Cassetta et al., 2017 [20]	Prospective controlled study	Italy/University clinic	14 pts; 20 teeth; 12–13 y	Tooth germ	Germectomy + orthodontics	Orthodontics only	Until LM2 eruption	Adjacent second molar outcomes; QoL
Mazur et al., 2025 [14]	Prospective observational	Italy/University clinic	51 adolescents; 87 teeth; 10–16 y	Demirjian B–D	Mandibular germectomy	None	7 days	Pain (VAS); PROMs
Ludovichetti et al., 2023 [18]	Randomized Clinical Trial	Italy/University clinic	20 Adolescents; 40 teeth; 11–16 y	Late germ/early root stage	Germectomy (flap comparison)	Alternative technique	7–14 days	Pain; swelling; trismus
Zhang et al., 2012 [10]	Retrospective cohort	China/Hospital	1050 pts; 808 teeth; mean 17 vs. 39 y	Immature vs. mature roots	Early extraction	Delayed extraction	Up to 6 months	Complications; IAN injury
Yunusov et al., 2025 [21]	Prospective comparative	Uzbekistan/University hospital	36 adolescents & young adults; 128 teeth; Group A 9–12 years, Group B 13–17 years	Early developmental stage	Early extraction/germectomy	Delayed extraction	Not specified	Complications; operative difficulty
Sivolella et al., 2011 [17]	Randomized Prospective Crossover	Italy/University clinic	26 Children; 52 teeth; mean age 15.4 years	1st Group (19 pts) with formed crown and no more than one third of the root 2nd Group (7 patients) with a crown and up to two thirds of the root	Bilateral mandibular germectomy	Alternative technique	Up to 1 month	Postoperative pain, complication, operative time
Chossegros et al., 2001 [11]	Prospective cohort	France, University hospital	154 adolescents; 300 teeth; mean 14.9 (12–19 years)	Developmental buds prior to anchoring of the roots in the jaw	Germectomy and bilateral germectomy	Alternative technique	15 days	Lingual nerve injury

**Table 2:** Post-operative complications.

Author (Year)	Study	Germectomy/Early Removal (n/N, %, Type)	Comparator/Delayed Extraction (n/N, %, Type)	Definition of IAN Injury/Duration
Chiapasco et al., 1995 [16]	Retrospective cohort	Group A (germectomy, n = 500): 13/500 (2.6%) total complications: secondary infection 10 (2.0%), severe trismus 2 (0.4%), excessive bleeding 1 (0.2%); no alveolar osteitis or IAN/LN paresthesia	Comparator Group C (≥24y, n = 430): 32/430 (7.4%): alveolar osteitis 9 (2.1%), secondary infection 8 (1.8%), excessive bleeding 4 (0.9%), IAN dysesthesia 6 (1.4%; 1 persistent at 25 months), severe trismus 3 (0.7%), 2nd molar restoration damage 2 (0.5%).	Dysesthesia: 1 persistent (25 months)
Monaco et al., 2017 [12]	Prospective cohort	Delayed-onset infection: 20/218 germectomies (9.2%), occurring 2–8 weeks post-op; clinically purulent exudate from the alveolus and swelling.	Not available	Not available
D’Angeli et al., 2021 [19]	Prospective cohort	Overall complications: 2/46 germectomies (4.3%): delayed-onset infection 1/46 (after 1 month) and postoperative bleeding 1/46 (immediately after surgery). No swelling/pain at 1 week; no alveolar osteitis, IAN/LN paresthesia, or trismus at subsequent follow-ups.	Not available	Not available
Cassetta et al., 2017 [20]	Prospective controlled study	No important adverse events or side effects recorded.	Not available	Not available
Mazur et al., 2025 [14]	Prospective observational	Not available	Not available	Not available
Ludovichetti et al., 2023 [18]	Randomized Clinical Trial	Not available	Not available	Not available
Zhang et al., 2012 [10]	Retrospective cohort	Early removal: total complications 20/808 (2.48%), nerve injury 0%	Delayed extraction: total complications 81/810 (10%), nerve injury 13/810 (1.6%), with 2 cases of permanent IAN numbness (>6 months).	Numbness: 2 persistent (>6 months)
Yunusov et al., 2025 [21]	Prospective comparative	Complications (overall): Group A 1/28 (3.6%); Group B 2/46 (4.3%); Group C 7/54 (13.0%). (Types not detailed in the table; narrative reports a case of secondary infection in Group A).	Not available	Not available
Sivolella et al., 2011 [17]	Randomized Prospective Crossover	Split-mouth (26 patients; 26 sites/arm): persistent swelling at 7 days 8/26 (30.7%) rotatory vs. 7/26 (26.9%) piezo; at 30 days 1/26 (3.85%) in both. Alveolar osteitis at 7 days 1/26 (3.85%) in both. Secondary infection at 30 days 1/26 (3.85%) rotatory vs. 0/26 piezo. Severe trismus 2/26 (7.7%) rotatory vs. 0/26 piezo. Postoperative bleeding, IAN/LN dysesthesia: 0% in both.	Not available	Not available
Chossegros et al., 2001 [11]	Prospective cohort	Randomized prospective: no subjective or objective evidence of transient or permanent lingual nerve injury-0/116 with lingual nerve protection and 0/138 without protection (254 procedures assessed at follow-up). Other postoperative complications not reported.	Not available	Not available

Note: “Not available” indicates either that the study was non-comparative and therefore did not include a delayed-extraction comparator arm, or that a comparator was present, but the original study did not report complication data for that specific outcome in sufficient detail.

**Table 3:** GRADE assessment of certainty of evidence for primary outcomes. Question: In pediatric or adolescent patients, does early mandibular third molar removal (germectomy/immature extraction) influence postoperative complications compared with delayed extraction?

Outcome (Meta-Analysis)	No. of Studies	Study Design	Risk of Bias	Inconsistency	Indirectness	Imprecision	Publication Bias	Effect (RR, 95% CI)
Inferior alveolar nerve (IAN) injury	2	Comparative cohort studies	Serious ^a^	Not serious	Not serious	Serious ^b^	Undetected ^c^	0.05 (0.01–0.37)
Overall postoperative complications (composite)	3	Comparative cohort studies	Serious ^a^	Not serious	Serious ^d^	Serious ^b^	Undetected ^c^	0.29 (0.20–0.42)

^a^ Downgraded for risk of bias due to non-randomised designs and concerns for confounding/baseline differences and incomplete outcome ascertainment in several included studies. ^b^ Downgraded for imprecision due to few studies and rare events (particularly for IAN injury), with wide confidence intervals and limited information size. ^c^ Publication bias could not be formally assessed because of the small number of studies (<10); therefore, it was considered undetected but remains possible. ^d^ Downgraded for indirectness because the definition of ‘overall complications’ varied across studies (composite endpoint) and follow-up windows for capturing late events were inconsistent.

## Data Availability

No new data were created or analyzed in this study.

## Supplementary Materials

The following supporting information can be downloaded at: https://www.mdpi.com/article/10.3390/dj14040215/s1. File S1: PRISMA 2020 Checklist.
